# Supplementary material for: PCR-Based Strategy for Introducing CRISPR/Cas9 Machinery into Hematopoietic Cell Lines
Source: Cancers (Basel). 2023 Aug 25;15(17):4263. doi: 10.3390/cancers15174263 (PMC10487029; doi:10.3390/cancers15174263)

Supplemental Table S1. Sequences of the sgRNA used to target IDH2 and MYBL2 and the ssODN sequence.

sgRNA	Sequence (5'-3')
MYBL2	TTGGCAGCTGGCTCCCTC
*IDH2*_1	TGGACCAAGCCCATCACCAT
*IDH2*_2	CTGGCCTACCTGGTCGCCAT
*IDH2*_R172K ssODN	tgcaaaaacatcccacgcctagtccctggctggactaaaccgataacgatcggaa**a** gcacgcgcatggcgaccaggtaggccagggtggagaggggat

Supplemental Table S2. Primers sequence for sgRNA constructs. sgRNA sequence should be added to Primer FusionPCR_1F and FusionPCR_2R sequence in 5′ (sgRNA forward sequence in FusionPCR_1F and sgRNA reverse sequence in FusionPCR_2R).

Name	Sequence (5'-3')
FusionPCR_1F	GTTTTAGAGCTAGAAATAGCAAG
FusionPCR_1R	CCGGCAGACGACGATTTATGACGGAAAAAAGCACCGACTCGGTGCCAC
FusionPCR_2F	AAAAAACCTCCCACACCTCC
FusionPCR_2R	GGTGTTTCGTCCTTTCCACAA
FusionPCR_3F	GTTGTGGTTTGTCCAAACTC
FusionPCR_3R	CCGGCAGACGACGATTTATGACGG
FusionPCR_4F	GGGTCATTAGTTCATAGCCC
FusionPCR_4R	GGTGTTTCGTCCTTTCCACAA
FusionPCR_5F	AGCCCATATATGGAGTTCCG

Supplemental Table S3. Primers used in PCR 1 for library preparation.

Off-target	Primer Forward (5'-3')	Primer Reverse (5'-3')
sgRNA1_1	GATGATTGCCATAGCCTCAG	ATCCTATCTCCCACACTGTG
sgRNA1_2	GTACTCTAGCCTGGTGACAG	AGAATGGGAGGGGGAATAAG
sgRNA1_3	AGAGGTCATCTGTGTCCAGG	CTGGGACTCGGCCTTCTGAA
sgRNA1_4	TGAAAGGAGGGTGAGCCG	GAAGGTTCCCCCCTCATTTG
sgRNA1_5	AGTATCACTGGCATCTCTGC	CTGAGGAGGCCAGAATGACT
sgRNA1_6	CCATATGGGAGGGCGGTAGC	CAGAACAGATTCCCCCACAG
sgRNA1_7	CCTACCAGTGGAGCCTTGAG	GCTTCGATCCTGTTCCCTCC
sgRNA1_8	TGTTGGACTAGATCACTCTG	GCCCTTAGAGGGTAAACAAC
sgRNA1_9	CGCCAGGTCTGCAATGGAAG	GCCTCTTGTGAACTCTCCTC
sgRNA1_10	GCCCTGTCAATGAAGTGAAG	CCACAAATGGCTGCAGAAAC

sgRNA1_11	AAGGCCCTGTCAATGAACTG	TTCACTGCTAGGTTTGCCAC
sgRNA1_12	GTTGGGATTACAGGTATGAG	TAGTCAAGGTCTTATTGCAC
On-target	TTATCTCTGTCCTCACAGAG	CAAAGTCTGTGGCCTTGTAC
sgRNA2_1	TGTGTGTGGTTTGGAGGATG	AGCCATTCTTCCTGTACAGC
sgRNA2_2	CTGAAAGCGTATAGCTATCC	GGCCTTGGGTATGAAATGAAG
sgRNA2_3	CCACTGTTGGTGTGAGGAAG	GCATGTCCCTCAACCTTACTC
sgRNA2_4	AGCCCTACTGTAATGCTTGG	ATGTCATCCATCAGTCCCCG
Adapter sequence for Forward primer	TACACTCTTTCCCTACACGACGCTCTTCCGATCT	
Adapter sequence for Reverse primer	GTGACTGGAGTTCAGACGTGTGCTCTTCCGATCT	

Supplemental Table S4. Primers used in PCR 2 for library preparation.

Name	Adaptador PCR2. Sequence (5' - 3')
**TruSeq Universal Adapter_ Forwar**	AATGATACGGCGACCACCGAGATCTACACTCTTTCCCTACACGACGCTCTTCCGATCT
**TruSeq_NB4_RNP-Ed**	CAAGCAGAAGACGGCATACGAGATGATCTGGTGACTGGAGTTCAGACGTGTGCTCTTCCGATCT
**TruSeq_NB4-Cas9-Ed**	CAAGCAGAAGACGGCATACGAGATTGGTCAGTGACTGGAGTTCAGACGTGTGCTCTTCCGATCT

Green nucleotides represent homolog sequence to primers for PCR 1. Indexes, which are tag sequences to mark each experiment, are showed as red nucleotides.

Supplemental Table S5. Primers used to amplify Cas9 cassette from hCas9 vector

Name	Sequence (5'-3`)
Cas9_F	TCTGCTCTGATGCCGCATAG
Cas9_R	CTGCGCAGATCTGCTATGGC

Supplemental Table S6. Primers used to amplify targeted region in *MYBL2* and *IDH2* for T7 and RFLP assays and ICE analysis.

Target	Forward sequence (5'-3')	Reverse sequence (5'-3')
MYBL2	CTTTCCCACCCTGTAGCAGG	CCACATGCTGCAAATGTCTCAG
IDH2	ACTCCAGAGCCCACACATTT	ATGCCGGGATTACAAGTGTC

Supplemental Table S7. Primers used to detect Cas9 lentiCRISPR V2 integration in cell lines.

Name	Sequence (5'-3')
Cell_Cas9_F	CGAGTACTTCACCGTGTATAACG
Cell_Cas9_R	CTCTTCGATCCGCTTCATTCTCT

Supplemental Table S8. Primers sequence for U6-sgRNA-terminator cassette amplification in PX458 vector. Recognition AflII site is indicated in italics.

Name	Sequence (5'-3)
pU6_sgRNA_F_AflII	AGCTGC*CTTAAG*GGTTCCTGGCCTTTTGCTGG
pU6_sgRNA_R_AflII	AGCTGC*CTTAAG*GGGTACCTCTAGAGCCATTTG

Supplemental Table S9. Results of NHEJ obtained with ICE analysis with different sg*IDH2* in HEK293 cells.

**Conditions**	**sg*IDH2*_1**	**sg*IDH2*_2**	sg*IDH2*_1 + sg*IDH2*_2	**250 ng PCR Cas9 sg*IDH2*_1 + sg*IDH2*_2**
**Exp. 1**	20	10	14	2
**Exp. 2**	10	16	44	1
**Exp. 3**	5	8	13	5
**Exp. 4**	0	0	13	-
**Average NHEJ (%±SEM)**	8.75±3.3	8.5±4.26	21±7.7	2.6±1.2

Supplemental Table S10. Results of NHEJ obtained with ICE analysis with different optimizations in HEK293 cells.

**Conditions**	**150 ng hCas9 7ng sg*MYBL2***	**250ng hCas9 12 ng sg*MYBL2***	**250 ng hCas9 35ng sg*MYBL2***	**250 ng hCas9 60ng sg*MYBL2***	**500 ng hCas9 23.3 ng sg*MYBL2***	**250 ng hCas9 35ng sg*MYBL2*-p**	**250 ng PCR Cas9 35ng sgMYBL2**	**PX458-MYBL2**
**Exp. 1**	8	16	0	18	12	8	0	8
**Exp. 2**	6	29	6	24	12	5	1	11
**Exp. 3**	13	11	13	18	20	17	0	14
**Exp. 4**	0	0	14	0	0	17	-	13
**Average NHEJ (%±SEM)**	6.75 ±2.7	14 ±6	8.25 ±3.3	15 ±5.2	11±4	11.75 ±3.1	0.3 ±0.3	11.5 ±1.3

Supplemental Table S11. NHEJ efficiencies obtained with ICE analysis in NB4-Cas9 cells using sg*IDH2* constructs or NB4 cells using RNPs.

Conditions	sgIDH2_1 + sgIDH2_2	RNPs
**Exp. 1**	10	78
**Exp. 2**	4	78
**Exp. 3**	0	67
**Average NHEJ (%±SEM)**	4.6±2.9	74.33±3.6

Original Images from this work

Figure 2B_original

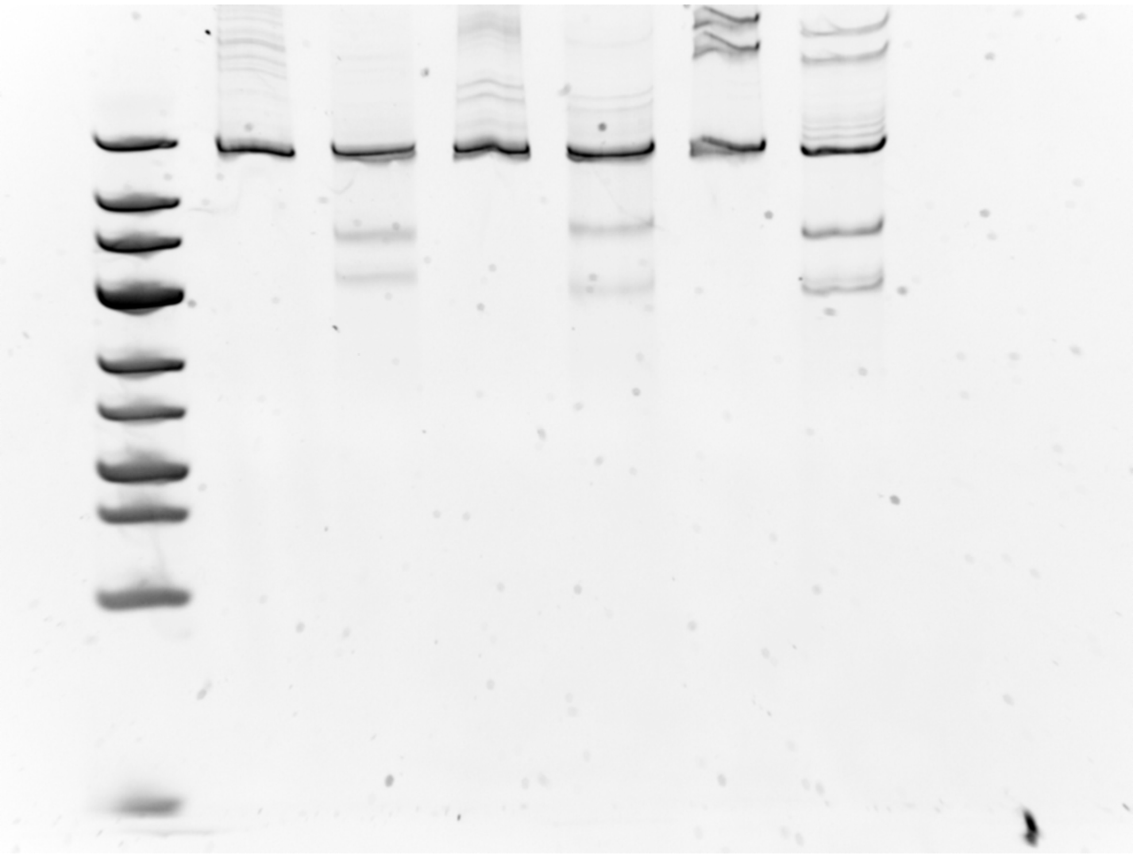

Figure 5A_original

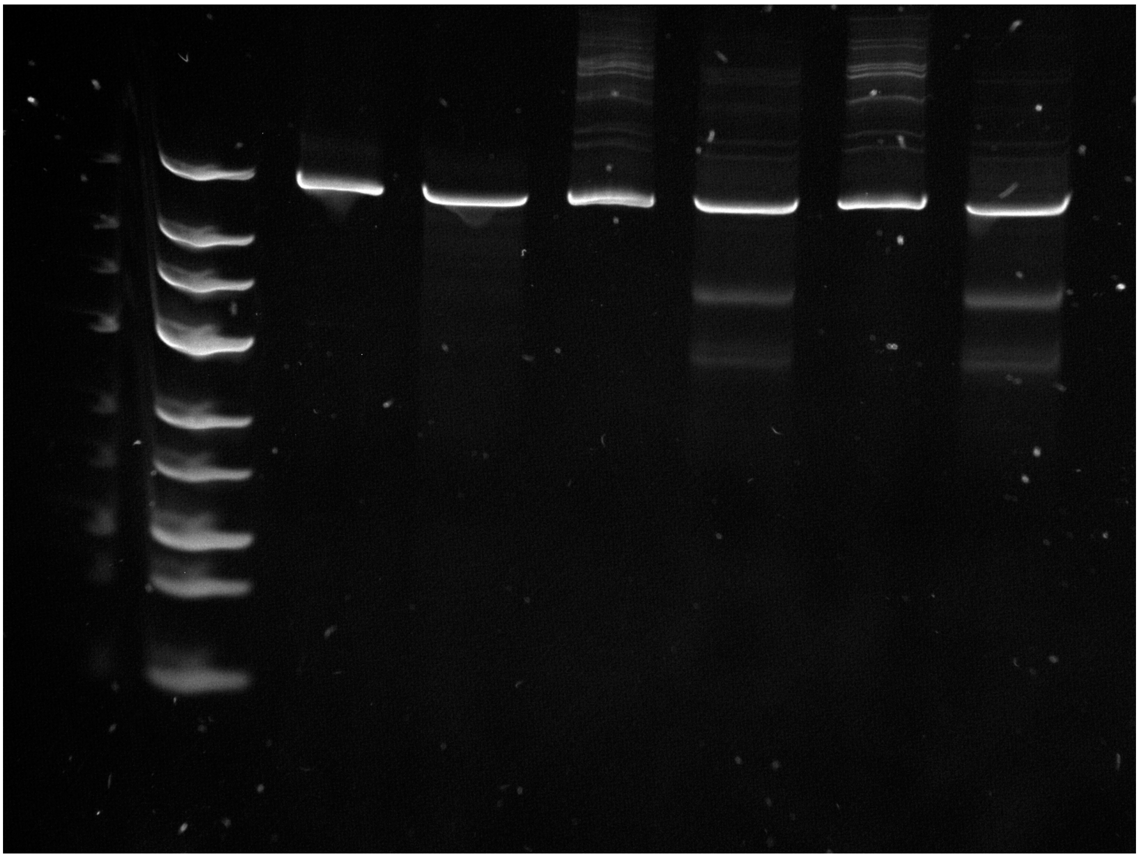

Figure 5B_original

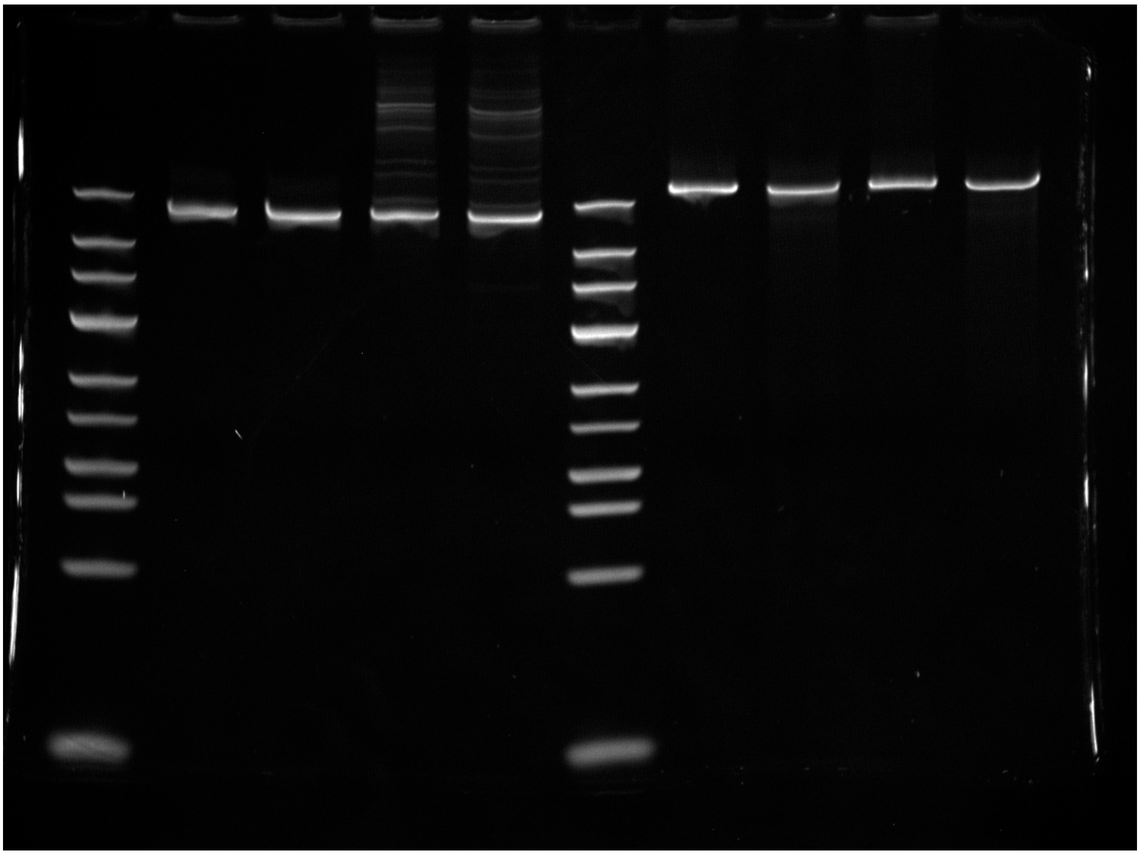

Figure 5C_original

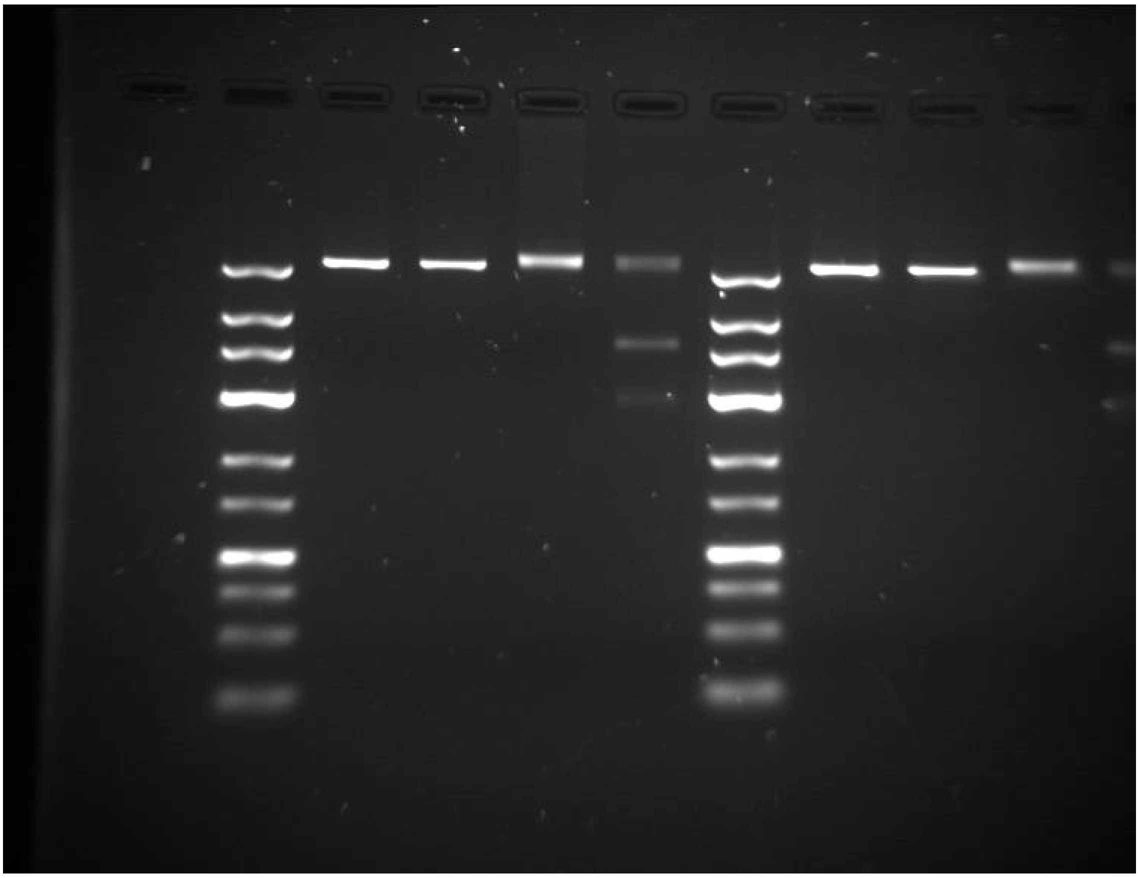

Figure 5D_original

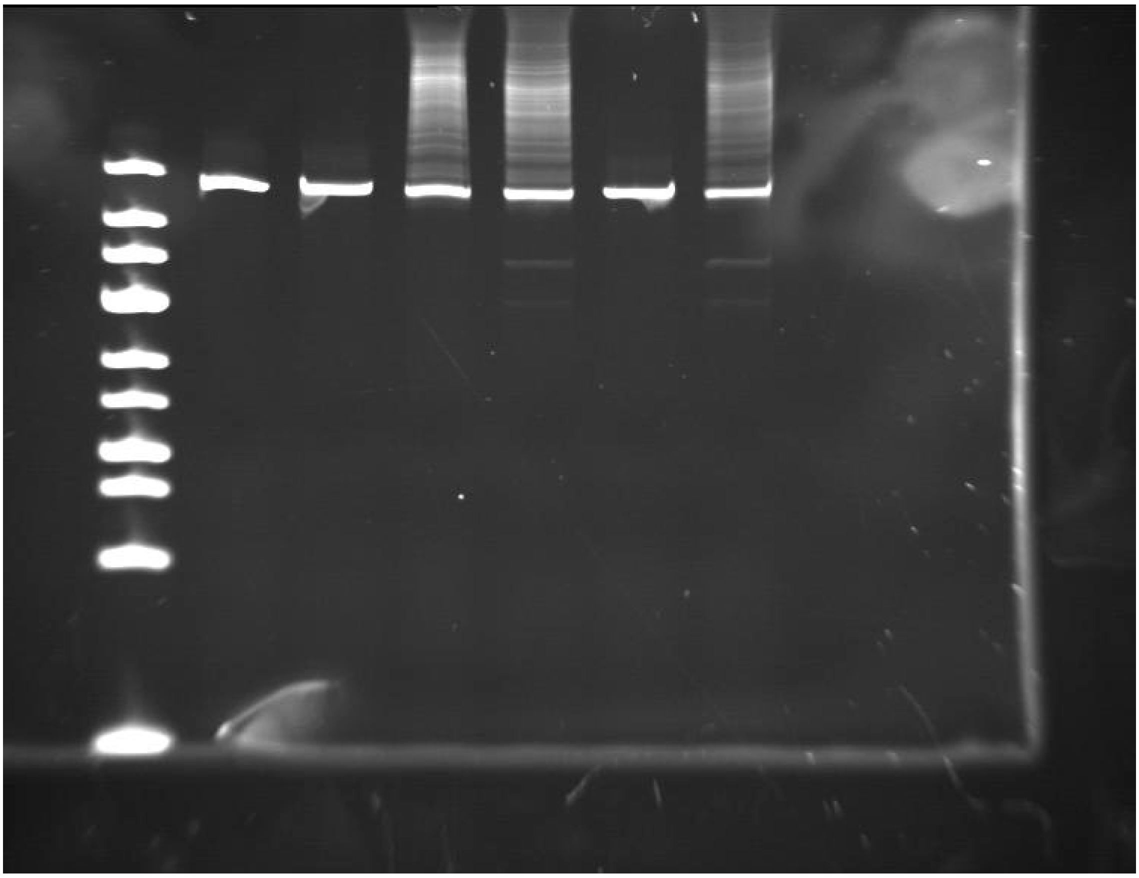

Supplement: Supplementary file 1 [file cancers-15-04263-s001.zip › cancers-2525037-supplementary.pdf]
